# Leprosy identified in Sumba Island, eastern Indonesia: elimination targets under threat

**DOI:** 10.1016/j.lansea.2024.100409

**Published:** 2024-04-23

**Authors:** Gladys O. Siregar, Maria Harianja, Jacklyn Adella, Hana Krismawati, Evivana S. Sundari, Messe R. Ataupah, Ruth D. Laiskodat, Claus Bøgh, Hardyanto Soebono, Marlous L. Grijsen

**Affiliations:** a Sumba Foundation, Sumba, Indonesia; b Center of Health System and Strategy, Ministry of Health, Jakarta, Indonesia; c Department of Dermatology and Venereology, Siloam Hospital, Kupang, Indonesia; d Province Health Office, East Nusa Tenggara, Kupang, Indonesia; e Department of Dermatology and Venereology, Gadjah Mada University, Yogyakarta, Indonesia; f Oxford University Clinical Research Unit Indonesia, Faculty of Medicine Universitas Indonesia, Jakarta, Indonesia; g Centre for Tropical Medicine and Global Health, Nuffield Department of Medicine, University of Oxford, UK

Leprosy is a neglected disease that primarily affects the skin, eyes, and nerves. More than 200,000 new cases of leprosy are reported each year worldwide.^1^ Although leprosy is curable, an estimated 3–4 million people globally live with some form of disability associated with leprosy.^2^ In 2000, WHO declared Indonesia to have successfully eliminated leprosy (prevalence of less than one case per 10,000 population).^3^ As a consequence, the national leprosy control programme was downscaled substantially, particularly affecting medically underserved areas.^4^ However, in recent years, 12,000–17,000 new cases have been reported annually, ranking Indonesia third in leprosy incidence only behind India and Brazil.^1^^,^^5^

Sumba is a remote island in eastern Indonesia with a population of 800,000, of which 29% live below the poverty line.^6^ Traditional small-scale subsistence farming is the predominant occupation. Sumba Foundation, a non-governmental organisation founded in 2001, supports five primary health clinics in West and Southwest Sumba and provides basic free-of-charge healthcare services.^7^ Even though around 15% of the outpatient consultations are skin-related, there is no dermatologist available in Sumba. The nearest dermatologist practices on a different island, a boat ride of more than 24 h away, limiting access to specialised skin care. In October 2020, we initiated a store-and-forward teledermatology programme for frontline healthcare workers (HCWs) in collaboration with Indonesian dermatologists who provided case-based support and training to improve access to skin care.^7^ Between October 2020 and March 2024, the programme has enabled HCWs to identify 60 people affected by leprosy, while in 2018 and 2019 only two new cases were reported by the local health authorities in the area.^8^

Among the 60 patients, 52 (87%) were newly diagnosed with leprosy. Seven (12%) individuals had previously been diagnosed with leprosy, but never completed treatment; one (2%) patient had completed treatment, but was suffering severe complications of the disease. According to the WHO-classification, 48 (80%) patients had multibacillary leprosy and 12 (20%) were paucibacillary (Table 1). Five (8%) patients were below the age of 18 years old. The median interval between onset of symptoms and diagnosis was 36 months (IQR 12–84 months). Twenty (33%) patients already had visible disabilities at diagnosis. Forty-three (72%) patients were identified during clinic visits (passive detection) and 17 (28%) during subsequent household screenings (active detection). Twenty (33%) individuals were part of family clusters (one cluster of eleven persons, one of four, one of three, and one of two persons), and four (7%) individuals were part of a village cluster (two clusters each consisting of two persons).

**Table 1:** Characteristics of the patients diagnosed with leprosy in West and Southwest Sumba between October 2020 and March 2024 (N = 60).

Patient characteristics	n (%)
**Female**	23 (38)
**Age** (years), median (IQR)	45 (30–53)
Children (<18 years)^a^	5 (8)
**Level of education**	
No education	17 (28)
Primary school	20 (33)
Secondary school	19 (32)
Tertiary school	4 (7)
**History of BCG vaccination**	
Yes	23 (38)
No	29 (48)
Unknown	8 (13)
**Type of leprosy**	
Paucibacillary	12 (20)
Multibacillary	48 (80)
**Ridley-Jopling classification**	
Borderline tuberculoid leprosy	19 (32)
Mid-borderline leprosy	4 (7)
Borderline lepromatous leprosy	16 (27)
Lepromatous leprosy	16 (27)
Indeterminate	3 (5)
Pure neural leprosy	1 (2)
Completed MDT, late stage complications	1 (2)
**Positive slit skin smear**^b^	42 (70)
BI-score, median (IQR)	2 (1–3)
**Physical disability (WHO criteria)**	36 (60)
Grade 1^c^	16 (27)
Grade 2^d^	20 (33)
**Leprosy reactions**^e^	8 (13)
Type 1 reaction	5 (8)
Type 2 reaction	3 (5)

Due to limited resources, we were not able to perform molecular testing to characterise the Mycobacterium species.

a Children were aged 5, 13, 16, 16, and 17 years at diagnosis.

b Slit-skin smears were taken from four sites: both earlobes and one or two active skin lesions.

c Loss of protective sensibility in eyes, hands or feet without visible damage or deformities.

d Presence of visible damage or deformities.

e Patients were diagnosed with a reaction at diagnosis (n = 5) or after initiation of multidrug therapy (n = 3).

The local health authorities have provided multidrug therapy (MDT) for all newly diagnosed patients who are seen monthly at one of the Sumba Foundation health clinics or at home. We experienced multiple challenges in providing prompt and uninterrupted treatment. First, essential drugs, including MDT, are typically dispensed from Ministry of Health to provincial health office, district health office, and finally to the primary health centres. These complex bureaucratic and logistical steps result in unnecessary delays in treatment initiation and treatment interruptions, which carry the risk of continued transmission, development of drug resistance and drop-out.^9^ Second, diagnostic and treatment delays were aggravated due to limited awareness and lack of clinical expertise and stigma among HCWs towards people affected by leprosy.^10^ Many of our patients had been referred from one healthcare centre to the other before the correct diagnosis was made. Third, leprosy reactions, which are (sub)acute episodes of increased inflammation, were misdiagnosed and undertreated, leading to preventable nerve damage and disfigurements. Last, undertaking community case finding efforts are challenged by poor infrastructure, low health literacy and travel time to reach the most remote communities.

Our experiences in Sumba illustrate the practical challenges associated with providing leprosy care in remote settings with limited resources, and how those translate into significant risks of forward transmissions and disabilities. We emphasise that leprosy, after reaching national elimination, can re-emerge in pockets of high endemicity. Our findings may be the ‘tip of the iceberg’, indicating a substantial unrecognised disease burden. The teledermatology programme shows the potential of telemedicine to improve access to specialised healthcare for hard-to-reach populations most in need.^11^ Establishing trust and collaboration with local and national government institutions are crucial to raise awareness and address the needs of persons affected by leprosy. To reinforce the global WHO-strategy ‘Towards Zero Leprosy’, our findings call for structural investments in case reporting, contact tracing, active case finding and improved access to diagnostics and treatment, especially in underserved communities. Further research is needed to understand the disease burden and develop interventions to promote access to prevention, identification, and management of leprosy for the people of Sumba, as well as across similar settings in Indonesia.

## Contributors

GOS, MH, JA, and CB are employed by Sumba Foundation and were involved in the clinical management and data collection of the persons affected by leprosy. ESS, HS and MLG are dermatologists and supported the teledermatology consultations. HS, CB and MLG were responsible for conceptualisation of the teledermatology programme and funding acquisition. HK, ESS, MRA, RDL, CB, HS and MLG were responsible for project administration. GOS, MH, JA, ESS, CB, HS and MLG take full responsibility for the integrity of the data and the accuracy of the data analysis. GOS and MLG drafted the manuscript. All authors provided valuable input and critically revised the manuscript. All authors reviewed and approved the final version of the manuscript. GOS, MH, JA, ESS, CB, HS and MLG had access to and verified the study data. HS, HK, CB and MLG were responsible for the decision to submit the manuscript for publication.

## Declaration of interests

The project was financially supported by the Wellcome Trust Africa Asia Programme Vietnam. The authors declare that they have no conflicts of interest.

## References

[1] Global leprosy (Hansen disease) update (2020). 2019: time to step-up prevention activities. Wkly Epidemiol Rec.

[2] Towards Zero Leprosy (2017).

[3] World Health Organization (2010). Progress in leprosy control: Indonesia, 1991–2008. Wkly Epidemiol Rec.

[4] Pieter Y., Grijsen M.L. (2022). Picturing health: the burden of leprosy in eastern Indonesia. Lancet.

[5] Global leprosy (Hansen disease) update (2023). 2022: new paradigm - control to elimination. Wkly Epidemiol Rec.

[6] Badan Pusat Statistik Provinsi Nusa Tenggara Timur Persentase penduduk miskin menurut kabupaten/Kota (persen) 2020-2022. https://ntt.bps.go.id/indicator/23/584/1/persentase-penduduk-miskin-menurut-kabupaten-kota.html.

[7] Adella F.J., Ammah H., Siregar G.O. (2024). Teledermatology to improve access to and quality of skin care in Eastern Indonesia. Am J Trop Med Hyg.

[8] Annual National Leprosy Report (2018). Directorate general of prevention and disease control, Ministry of Health, Republic of Indonesia.

[9] Weiand D., Thoulass J., Smith W.C. (2012). Assessing and improving adherence with multidrug therapy. Lepr Rev.

[10] Haverkort E., van ‘t Noordende A.T. (2022). Health workers' perceptions of leprosy and factors influencing their perceptions in endemic countries: a systematic literature review. Lepr Rev.

[11] Nelson C.A., Kovarik C.L., Morssink C.B. (2014). Tele-leprology: a literature review of applications of telemedicine and tele-education to leprosy. Lepr Rev.

